# CPDC-MFNet: conditional point diffusion completion network with Muti-scale Feedback Refine for 3D Terracotta Warriors

**DOI:** 10.1038/s41598-024-58956-1

**Published:** 2024-04-09

**Authors:** Xueli Xu, Da Song, Guohua Geng, Mingquan Zhou, Jie Liu, Kang Li, Xin Cao

**Affiliations:** 1 School of Information Science and Technology, Northwest University, Xi’an, 710127 Shaanxi China; 2 Yan’an University, Yan’an, 716000 Shaanxi China; 3 National and Local Joint Engineering Research Center for Cultural Heritage Digitization, Xi’an, 710127 Shaanxi China; 4 College of Computer and Information Engineering, Henan Normal University, Xinxiang, 453007 Henan China; 5 Big Data Engineering Laboratory for Teaching Resources & Assessment of Education Quality, Xinxiang, 453007 Henan China

**Keywords:** Applied optics, Computer science, Information technology

## Abstract

Due to the antiquity and difficulty of excavation, the Terracotta Warriors have suffered varying degrees of damage. To restore the cultural relics to their original appearance, utilizing point clouds to repair damaged Terracotta Warriors has always been a hot topic in cultural relic protection. The output results of existing methods in point cloud completion often lack diversity. Probability-based models represented by Denoising Diffusion Probabilistic Models have recently achieved great success in the field of images and point clouds and can output a variety of results. However, one drawback of diffusion models is that too many samples result in slow generation speed. Toward this issue, we propose a new neural network for Terracotta Warriors fragments completion. During the reverse diffusion stage, we initially decrease the number of sampling steps to generate a coarse result. This preliminary outcome undergoes further refinement through a multi-scale refine network. Additionally, we introduce a novel approach called Partition Attention Sampling to enhance the representation capabilities of features. The effectiveness of the proposed model is validated in the experiments on the real Terracotta Warriors dataset and public dataset. The experimental results conclusively demonstrate that our model exhibits competitive performance in comparison to other existing models.

## Introduction

The Terracotta Warriors of Qin Shi Huang is a cultural treasure of China and an important archaeological source for ancient Chinese science, culture, military, and other fields. Due to the long history and difficulty of excavation, the Terracotta Warriors often have different degrees of damage, and the restoration plan of the Terracotta Warriors has always been a hot topic of cultural relic protection. Manual restoration of the missing areas of the Terracotta Warriors usually faces the problems of large workload, high difficulty, and low efficiency. Using digital technology to restore cultural relics can effectively solve these problems and reduce the damage to the cultural relics themselves. The traditional Terracotta Warriors’ completion methods are mainly divided into template-based matching and grid surface fitting methods. The former method matches the most suitable template from the template library to repair the hole, while the latter method fits and reconstructs the hole area using the topological relationship of the 3D mesh. These methods are computationally expensive and cannot handle 3D models that are large and have many holes.

With the rapid development of deep learning, many learning-based methods (such as Pcn^1^, Topnet^2^, and Grnet^3^) are proposed to recover the complete shape by inferring the missing parts. These methods typically employ the Chamfer Distance (CD) or Earth Mover’s Distance (EMD) as loss functions to measure the dissimilarity between the generated complete point cloud and the ground truth. However, CD loss is not sensitive to overall density distribution and EMD loss is too expensive to compute in training. Generative adversarial network (GAN)^4^ is a generative method based on adversarial training, consisting of a generator and a discriminator. The generator produces images from a random vector, while the discriminator distinguishes between real and generated data. GAN’s advantages are that it can quickly generate images in a discrete pixel space and improve the quality and diversity of generated images through different loss functions and regularization methods. However, GAN’s training process may be unstable, leading to mode collapse or low-quality output. Additionally, GAN requires careful tuning of hyperparameters and loss functions to achieve good results, which can be time-consuming and difficult.

Recently, probabilistic diffusion models, a novel family of generative models, have demonstrated remarkable results in generating 2D images and 3D point clouds^5–9^. These approaches train a probabilistic model to simulate a denoising process. Diffusion is guided to progressively transform a Gaussian noise into a target output. Diffusion probabilistic models have a more stable training procedure and a better generation quality than GANs, which can be trained with a simple loss function. Lyu^6^ finds that Denoising Diffusion Probabilistic Models (DDPM)^5^ can generate uniform and high-quality point clouds, using an efficient and effective loss function. They introduce the Point Diffusion-Refinement (PDR) paradigm for point cloud completion, which also leads to a simultaneous improvement in generation speed. However, PDR directly handles the 3D point cloud and uses a complex Condition Feature Extraction subnet, which leads to huge network computation. Luo^10^ is the first work to apply DDPM to the problem of unconditional point cloud generation, where the goal is to generate realistic point clouds from noise without any guidance. Zhou et al.^11^ introduce Point-Voxel Diffusion (PVD), a probabilistic and flexible shape generation model that addresses the above challenges by combining denoising diffusion models with the hybrid point-voxel representation of 3D shapes, which enables the synthesis of high-fidelity shapes and the completion of partial point clouds. These methods can generate diverse and high-quality results.

Inspired by PDR^6^, we present a conditional point diffusion completion network with a multi-scale refinement network (CPDC-MFNet) model to expedite the Terracotta Warrior point cloud completion process. Meanwhile, we introduce an innovative sampling algorithm aiming at enhancing the precision of our generative model by effectively aggregating localized information. Furthermore, a probabilistic model based on diffusion is proposed for the completion of Terracotta Warriors’ point cloud. The model can infer the conditional probabilities of the position changes of each particle during the diffusion process from the observed incomplete Terracotta Warrior fragments, and use these conditional probabilities to generate new complete models. To achieve this, we use the Markov chain to model the reverse diffusion process that transforms the noise distribution to the target distribution. However, the Markov chain only models the point distribution and cannot create point clouds with different shapes on its own. For this reason, we add a shape latent variable as the condition for the transition kernel. When generating point clouds, the shape latent variable has a prior distribution that we parameterize with normalizing flows for high model flexibility. When auto-encoding point clouds, the shape latent variable is added to the network. Our training objective is to maximize the variational lower bound of the likelihood of the Terracotta Warrior point cloud given the shape latent variable, which can be written in a simple form. To extract feature information more quickly and effectively, we propose a new sampling algorithm Partition Attention Sampling (PAS) to aggregate local information. Simultaneously, to address the issue of slow sampling in DDPM, we introduce a multi-scale refine network to accelerate the generation process. Extensive experiments on the real-world Terracotta Warriors dataset and the public dataset (ShapeNet) are conducted. The results show that our model can perform well on point cloud completion and is competitive on this task.

Our main contributions can be summarized as:

1. We propose a probabilistic model based on diffusion for Terracotta Warriors point cloud completion. The model can infer the conditional probabilities of the position changes of each particle during the diffusion process from the observed incomplete point cloud, and use these conditional probabilities to generate complete point clouds.
2. We propose a multi-scale refine network (MSFR) model to accelerate the generation process.
3. We propose a new sampling algorithm Partition Attention Sampling (PAS) to aggregate local information effectively and efficiently.
4. We also demonstrate the effectiveness of CPDC-MFNet on real-world scans and public dataset.

## Experiments and results

### Datasets

The data of the Terracotta Warriors are collected by the visualization laboratory, with a total of 78 Terracotta figures which are acquired by using Creaform VIU 718 hand-held 3D scanners. Furthermore, the Terracotta Warriors are unearthed from the K9901 pit of Emperor Qinshihuang’s Mausoleum Site Museum. The scan resolution is 0.05 mm, which is conducive to scan speed. First, we use Geomagic Design software to separate Terracotta Warriors mesh into different parts of the body. Then we use Blender software to randomly partition the Terracotta Warriors into 20 no-overlapping pieces. One to four parts of them are randomly selected as the missing part, and the remaining portion constitutes data that needs to be completed. We divide the dataset into three categories: (Arm: 91, Body: 60, and Leg: 80). Among them, 188 models are used for training (Arm: 74, Body: 50, Leg: 64), the left 43 models are used for testing (Arm: 17, Body: 10, Leg: 16). All the input point clouds are normalized to [− 1, 1].

### Evaluation metrics

To evaluate the accuracy of completed point clouds on our datasets, we use Chamfer Distance (CD), and Earth Mover’s Distance(EMD) as evaluation metrics. CD is defined in Eq. (1), where $|V|$ means the number of points in V. The former part measures the distance between the generated point cloud and the ground truth point cloud, and the latter part measures the coverage of the ground truth point cloud in the generated point cloud. The EMD is used to measure the shape discrepancy between the predicted point cloud V and the ground truth point cloud X, both of which have the same size N. It estimates a bijection distance between V and X. EMD is defined in Eq. (2).

1 $${\mathcal{L}}_{CD} \left( {V,X} \right) = \frac{1}{\left| V \right|} \mathop \sum \limits_{v \in V} \mathop {\min }\limits_{x \in X} \left| {\left| {v - x} \right|} \right|^{2} + \frac{1}{\left| X \right|} \mathop \sum \limits_{x \in X} \mathop {\min }\limits_{v \in V} \left| {\left| {x - v} \right|} \right|^{2}$$

2 $${\mathcal{L}}_{EMD} \left( {V,X} \right){ } = { }\mathop {\min }\limits_{\emptyset :V \leftrightarrow X} \mathop \sum \limits_{v \in V} \left| {\left| {v - \emptyset \left( v \right)} \right|} \right|^{2}$$

### Training setting

For the diffusion model, we adopt the PVCNN^12^ styled U-Net which is proposed in PVD^11^ to train our diffusion model. Following DDPM, the variance schedules to be  $\beta_{1}$ = 0.0001 and  $\beta_{{\text{T}}}$ = 0.05, and  $\beta_{{\text{t}}}$ (1 < t < T) is linearly interpolated, and the number of sample steps is 1000. We use a batch size of 32 and a learning rate of $2e^{ - 4}$. Since our approach is probabilistic, we compare it with two distribution-fitting models Point-Flow^13^ and PVD. We evaluate our model on three categories: arm, body, and leg with 5% missing, 10% missing, 15% missing, and 20% missing respectively. In the case of 5% missing, we conduct experiments at different resolutions, with 2048 points, 4096 points, and 8192 points, respectively.

## Results

**Figure 1:**
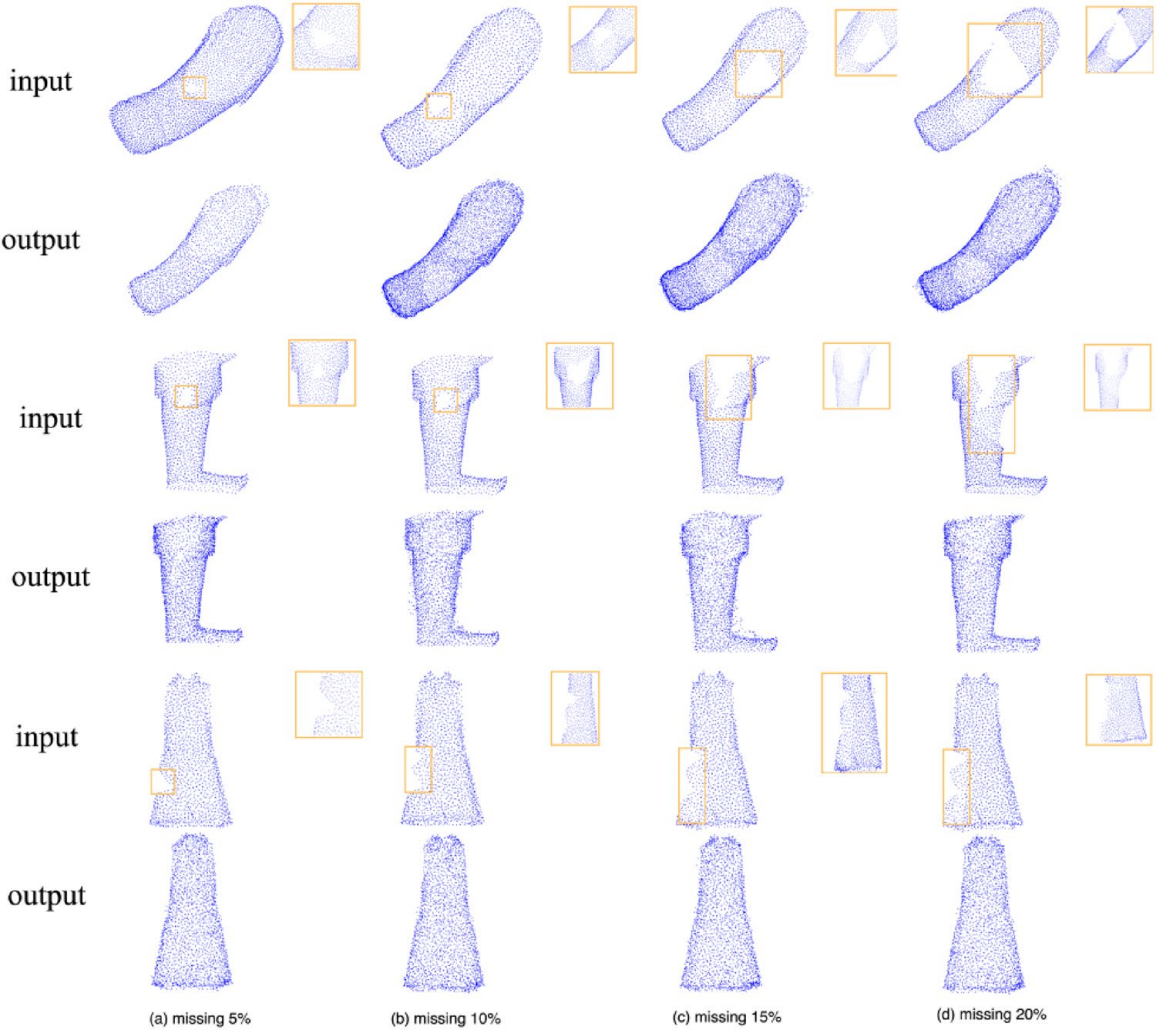
Completion results under different missing ratios at the resolution of 2048.

We conduct a series of experiments to evaluate our model. As the proportion of missing parts increases, the generation effect gradually deteriorates, as shown in Table 1. Across all three datasets, the most optimal experimental outcomes are consistently achieved when the missing parts constitute 5% of the whole. Worth noting, all the experiments depicted in Table 1 are executed at a resolution of 2048. In Fig. 1, we provide visual comparisons that offer a compelling insight into the generated results. In Fig. 1, the first, third, and fifth rows are the incomplete inputs, while the second, fourth, and sixth rows are the corresponding completion results. Among the two indicators, CD exhibits the highest sensitivity to variations in the percentage of missing parts.

**Table 1:** Quantitative comparison on the Terracotta Warriors dataset at the resolution of 2048 points.

Dataset	5% missing		10% missing		15% missing		20% missing	
	CD	EMD	CD	EMD	CD	EMD	CD	EMD
Arm	4.07	7.68	4.35	8.19	4.83	8.42	5.29	10.92
Body	2.02	4.54	2.54	2.99	2.20	4.54	2.67	5.67
Leg	4.43	8.91	4.79	9.59	5.19	11.47	5.81	11.62

**Figure 2:**
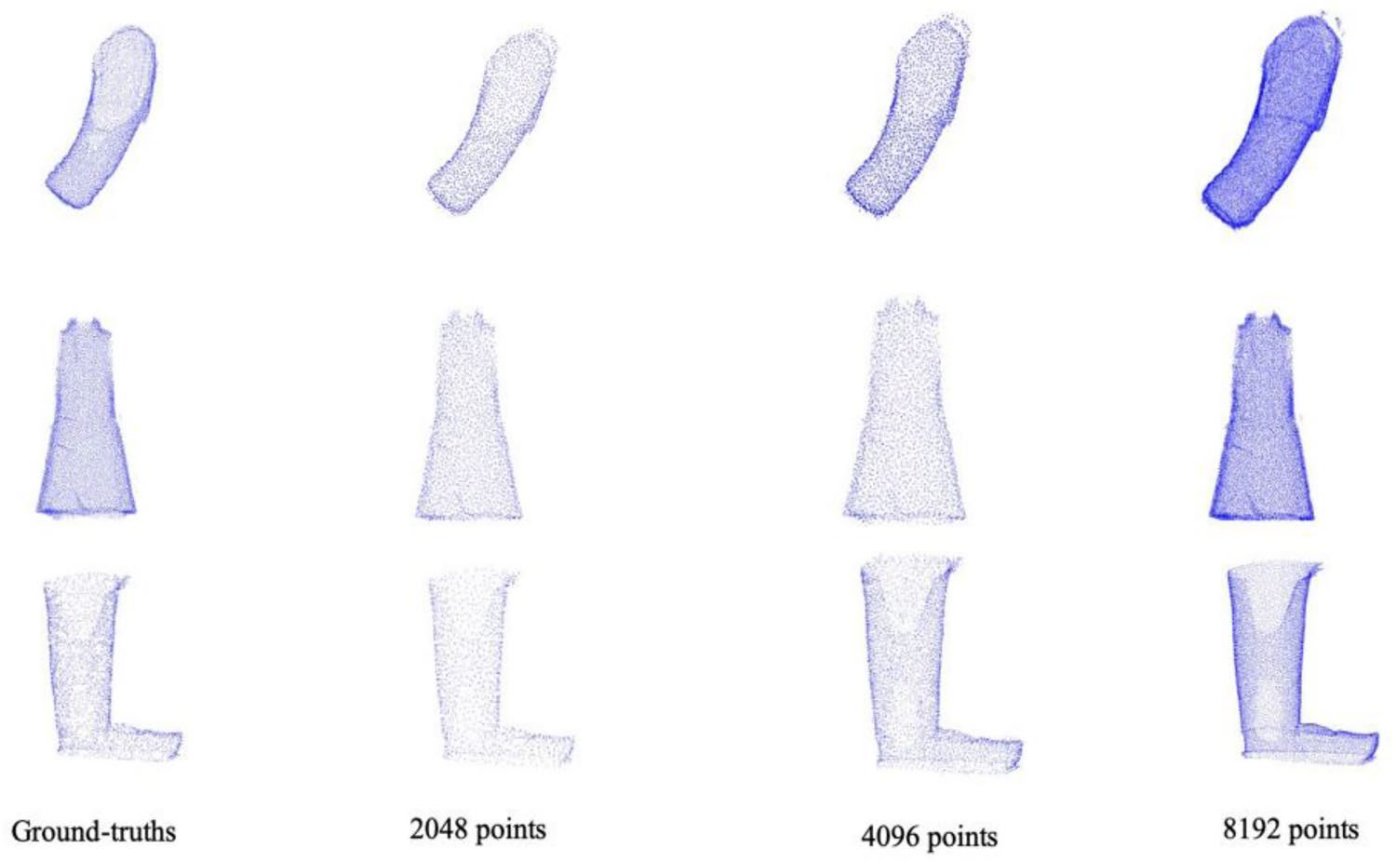
Completion results at different resolutions with the missing percentage of 5%.

We extend our examination to the completion results at various resolutions while keeping the proportion of missing data fixed at 5%. The outcome of these experiments, as presented in Table 2 (the visualization shown in Fig. 2), reveals an interesting trend: there is no significant variation in the results as the resolution adjusted. This observation suggests that our model's performance remains consistently robust across different levels of detail. As the number of point clouds increases, we reduce the size of the patch. The increase of points’ number does not improve the experimental results. Instead, it leads to a reduction in both generation and training time. The maximum average difference in the CD index at different resolutions is a mere 0.32, signifying that setting the resolution to 2048 is an appropriate choice. This consistency in performance across resolutions underscores the effectiveness of our methodology and highlights the efficiency of the selected resolution for our specific application.

**Table 2:** Quantitative comparison on the Terracotta Warriors dataset at the different resolution with the missing percentage of 5%.

Dataset	2048		4096		8192	
	CD	EMD	CD	EMD	CD	EMD
Arm	4.07	7.68	4.27	8.51	4.31	8.63
Body	2.02	4.54	2.43	4.86	2.52	4.85
Leg	4.43	8.91	4.50	9.00	4.47	8.94

**Figure 3:**
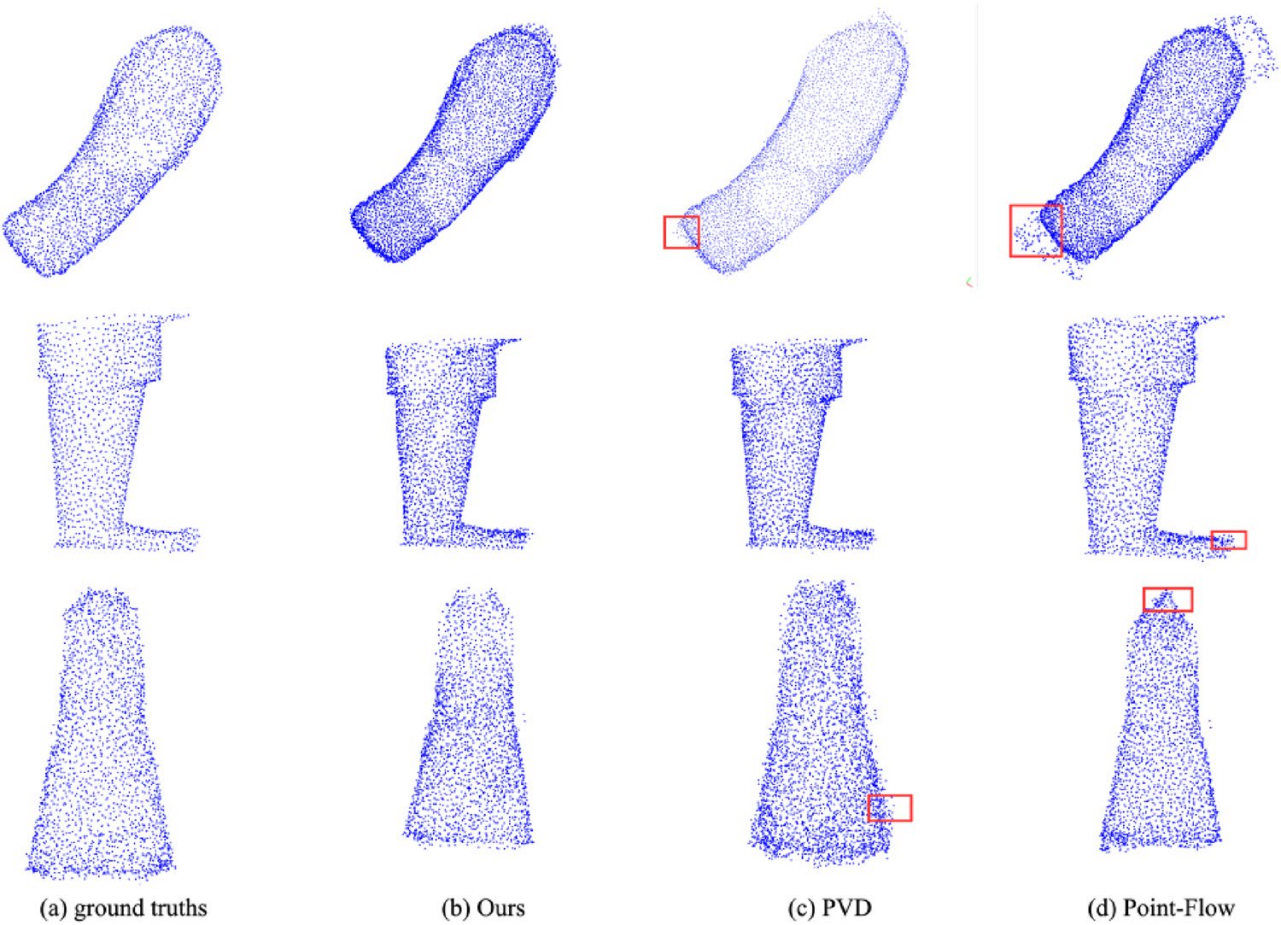
Comparing the point set completion results produced by PVD and Point-Flow at the resolution of 2048 with the missing percentage of 5%.

To evaluate the effectiveness of our approach, we conducted a comparative analysis with two probabilistic generation models: Point-Flow and PVD. The results of this evaluation are presented in Table 3. The table reveals that our method acquires comparable results with PVD and a greater advance than Point-Flow. However, the superiority of our approach becomes even more evident when we consider the visual quality of the generated output. Figure 3 showcases this distinction, emphasizing that our method consistently produces point clouds with clearer boundaries. The obvious redundancy points are framed in red in Fig. 3. This enhanced clarity is of significant importance, particularly in scenarios where the subsequent reconstruction into other data formats relies heavily on the precision of the generated results.

**Table 3:** Quantitative comparison with PVD and Point-Flow at the resolution of 2048 with the missing percentage of 5%.

Methods	Arm		Body		Leg	
	CD	EMD	CD	EMD	CD	EMD
Point-Flow	6.32	11.80	3.73	7.46	5.37	10.76
PVD	4.11	8.02	2.24	4.48	4.51	9.03
Ours	**4.07**	**7.68**	**2.02**	**4.54**	**4.43**	**8.91**

Significant values are in bold.

The results of comparison with other methods on the ShapeNet dataset are shown in Table 4. From Table 4, we can observe that we have achieved competitive results in EMD. According to Zhou^11^, better EMD scores are more indicative of higher visual quality, and CD is blind to visual inferiority. Therefore, our model has better visual results. Consequently, the favorable EMD scores achieved by our model reinforce the assertion that our method not only excels in quantitative measures but also translates into visually superior results compared to alternative approaches.

**Table 4:** Quantitative comparison with other methods on the ShapeNet dataset.

Category	Model	CD	EMD
Airplane	SoftFLow	0.4042	1.198
	PointFlow	**0.4030**	1.180
	DPF-NET	0.5279	1.105
	PVD	0.4415	1.030
	Ours	0.4671	**1.011**
Chair	SoftFLow	2.786	3.295
	PointFlow	**2.707**	3.649
	DPF-NET	2.763	3.320
	PVD	3.211	2.939
	Ours	3.109	**2.901**
Car	SoftFLow	1.850	2.798
	PointFlow	1.803	2.851
	DPF-NET	**1.396**	2.318
	PVD	1.774	**2.146**
	Ours	1.811	2.252

Significant values are in bold.

### Ablation studies

To validate the effectiveness of the PAS module and MSFR in our method, we implement a group experiments for the ablation study. The experiments are conducted on real Terracotta Warrior datasets at the solution of 2048 points of 5% missing and results are presented in Table 5. The results show that our model using both PAS and MSFR achieves the best results in two indicators. The CD index increased to 4.24, 2.46, and 4.65 in three categories, respectively, when PAS is removed. Removing MSFR, the CD index degenerates to 4.30, 2.07, and 4.51 in three categories, respectively. The results prove that the PAS and MSFR modules can effectively boost the reconstruction result.

**Table 5:** Ablation study for different components at the resolution of 2048 with the missing percentage of 5%.

Category	PAS	MSFR	CD	EMD
Arm	✓	✓	4.07	7.68
	⨉	✓	4.24	7.81
	✓	⨉	4.30	7.69
	⨉	⨉	4.51	7.92
Body	✓	✓	2.02	4.54
	⨉	✓	2.46	4.92
	✓	⨉	2.07	4.57
	⨉	⨉	2.58	5.12
Leg	✓	✓	4.43	8.91
	⨉	✓	4.65	9.21
	✓	⨉	4.51	9.08
	⨉	⨉	4.73	9.31

### Model accelerate

To validate the effectiveness of the MSFR network in our method, we implement a group of experiments for the ablation study. These experiments are carried out at resolution of 2048 points and 5% missing. The results are shown in Table 6. Note that in the case of 1000 sample steps, we do not use MSFR to refine the output. The results indicate that reducing the sampling steps to 200 results in only a minor decrease in the arm and leg datasets, but an improvement in the body dataset. The reconstruction results show a significant decrease until the sampling steps are reduced to 50. The experimental results show that MSFR can effectively reduce sampling steps while ensuring the generation quality does not decrease.

**Table 6:** Refine coarse point clouds generated by the DDPM at the resolution of 2048 points with the missing percentage of 5%.

Number of sample steps	Arm		Body		Leg	
	CD	EMD	CD	EMD	CD	EMD
1000	3.77	6.64	2.42	4.83	4.22	8.45
200	4.07	7.68	2.02	4.54	4.43	8.91
50	7.08	17.11	7.61	23.60	8.75	19.53

## Conclusion

In this paper, we propose the Conditional Point diffusion completion network with Muti-scale Feedback Refine network for Terracotta Warriors. It has achieved good results in completing the real Terracotta Warriors dataset. Our MSFR network effectively addresses the slow sampling speed issue of DDPM. By reducing the number of samples in the diffusion stage and optimizing the coarse point cloud generation, we achieve faster and more efficient generation results while maintaining high-quality. At the same time, the PAS module can effectively capture local feature information, enhancing the overall completion results. We believe that our network structure has the potential to be applied to other tasks. Our model has achieved competitive results on both the Terracotta Warriors dataset and the public dataset, and can reduce the number of samples by five times.

However, there are limitations that our method struggles with to predict salient points and small irregular surfaces. Addressing these challenges remains a key focus for future research and development. In the future, we plan to explore the application of diffusion models in latent spaces to generate richer completion results and to apply our structure to the class conditional generation task of Terracotta Warriors.

## Related work

### Point cloud completion

Point cloud generation is an essential task for many 3D vision tasks, such as filling in missing parts, increasing resolution, creating new shapes, and augmenting data. Following the lead of PointNet^14^, some works^1,2^ concentrate on learning global feature representations from 3D point clouds for generation, which however fail to capture fine and detailed shape features. To generate point clouds, some early methods adopt the approach of representing point clouds as matrices of $N\times 3$ dimensions^15,16^, where $N$ is the predetermined number of points in the point cloud. Through this approach, they transform the point cloud generation problem into a matrix generation problem, which enables them to apply existing generative models more easily. L-GAN^16^ is the first deep generative model for point clouds. Although it can perform shape completion tasks to some extent, its architecture is not primarily designed for this purpose, and therefore its performance is not considered ideal. FoldingNet^17^ introduces a decoding operation called Folding, which serves as a 2D-to-3D mapping. Subsequently, Point Completion Network (PCN) proposed in Yuan’s work^1^, is the first learning-based architecture that focuses on shape completion tasks and utilizes the Folding operation to approximate a relatively smooth surface for shape completion. These methods have a major drawback that they can only generate point clouds with a fixed number of points, and they lack the property of permutation invariance. Lately, a new viewpoint has emerged, suggesting that point clouds can be seen as samples drawn from a point distribution, such as these related works^13,16,18–20^. This perspective encourages the investigation of the application of likelihood-based techniques to the modeling and generation of point clouds, often yielding excellent outcomes.

### Diffusion probabilistic models

The diffusion process considered in this work is related to the diffusion probabilistic model^5,21^, which is a type of latent variable model that can generate data from noise. Diffusion probabilistic models are a class of latent variable models, which also use the Markov chain to convert the noise distribution to the data distribution. The diffusion model has been applied to various tasks. Baranchuk and Graikos^22,23^ use diffusion models in image segmentation, Zimmermann et al.^24^ explore the application of DDPM in the classification task, and other works^25–27^ use diffusion models in image super-resolution. These models utilize the Markov chain to transform the noise distribution into the data distribution in a series of steps. Because the Markov chain considered in our work generates points of a point cloud conditioned on some shape latent, which can be learned from data, in this work, we focus on the Terracotta Warriors point cloud completion, which is a conditional generation problem.

Luo et al.^10^ use a Point-wise net as their generator network, which is similar to a 2 stage PointNet that has been used for point cloud part segmentation tasks. However, the Point-wise net has a limitation that it can only receive a global feature as input. It cannot leverage fine-grained local structures in the incomplete point cloud, which are important for capturing the shape details and diversity. Zhou et al.^11^. extend the conditional DDPM framework to the problem of point cloud completion, where the goal is to generate a complete point cloud from an incomplete one. Zhou et al. train a point-voxel CNN^12^ as their generator network, which takes both the incomplete point cloud $c$ and the noisy input ${x}_{T}$ as input. However, their way of using $c$ is different from ours. Zhou et al. simply concatenate $c$ with ${x}_{T}$, and feed the concatenated point cloud to a single point-voxel CNN. This may degrade the performance of the network, because the concatenated point cloud may not have a uniform density or distribution. Moreover, ${x}_{t}$ becomes very different from $c$ as $t$ increases, due to the large noise magnitude in ${x}_{t}$. Feeding two point clouds with very different properties to a single network at once could confuse the network and make it hard to learn meaningful features. Zhao et al.^6^ train an additional refine network to accelerate sampling speed and improve generation efficiency. In our work, we draw inspiration from this approach.

### Feedback mechanism

The feedback mechanism allows the network to gain information from previous states. With feedback connections, high-level features are rerouted to the low layer to refine low-level feature representations. The feedback mechanism has been widely employed in various 2D image vision tasks, some works^28–30^ use feedback mechanism in image super-resolution, Sam^31^ and Feng^32^ use it to enrich network features, and Chen^33^ use it in image deraining problems. In the 3D field, Su^34^ and Yan^35^ use it to complete the point cloud. In our work, we use a feedback mechanism to refine our generation and accelerate the generation speed. Based on the feedback mechanism, completion results are optimized by multiple iterations to get the final refined result.

## Methods

**Figure 4:**
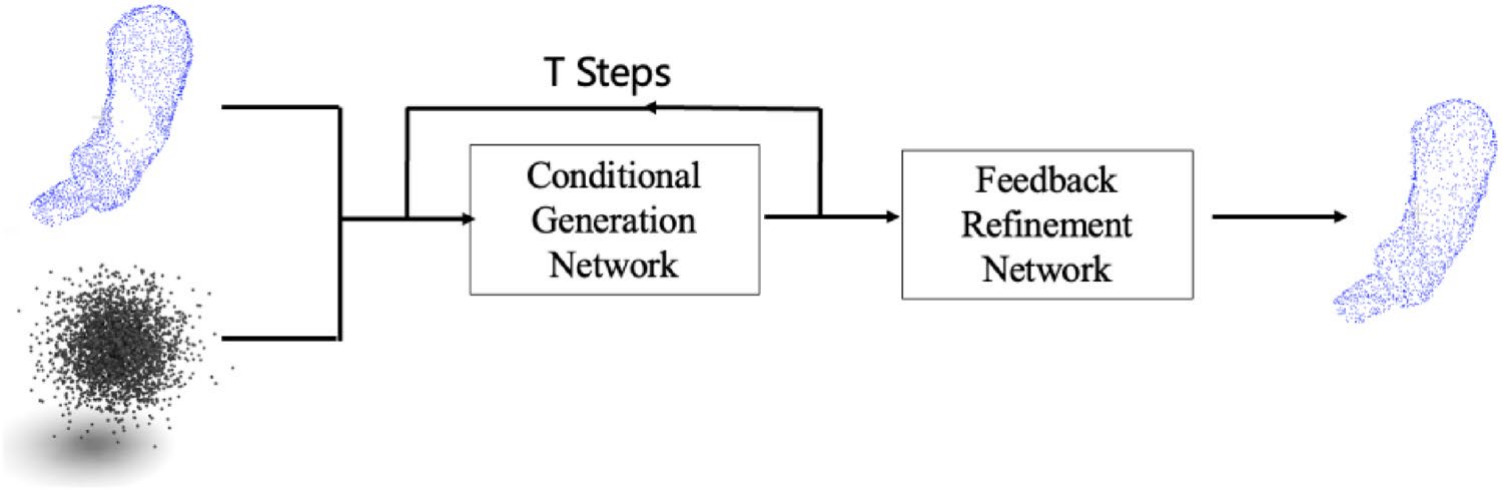
The pipeline of our network.

An overview of the conditional DDPM formulation is started, which is a generative model that can produce a completed point cloud from random noise. The overall pipeline of our network is shown in Fig. 4, which includes two modules, the conditional generation network with Partition Attention Sampling and a multi-scale refine network. The details will be described in the following sections.

### Formulation

The denoising diffusion probabilistic model is a type of generative model that models generation as a process of removing noise. It starts with Gaussian noise and performs denoising until a high-resolution shape emerges. Specifically, we assume that ${p}_{data}$ is the distribution of the whole point cloud ${x}_{i}$ in the dataset, and $p_{latent}$ = $N\left( {0_{3N} ,{ }I_{3N \times 3N} } \right)$ is the latent distribution, where N represents the Gaussian distribution. Then, the conditional DDPM is composed of two Markov chains named the diffusion process and the reverse process.

The diffusion process is a Markov process that adds Gaussian noise into the clean data $p_{data}$ until the output distribution is close to $p_{laten}$. The diffusion process is irrelevant to the conditioner, the incomplete point cloud $c_{i}$. The diffusion process from clean data $x_{0}$ to $x_{T}$ is defined as

3 $$q\left( {x_{1:T} {|}x_{0} } \right) = {\Pi }_{t = 1}^{T} q\left( {x_{t} {|}x_{t - 1} } \right),\;q\left( {x_{t} {|}x_{t - 1} } \right) = N\left( {x_{t} {|}\sqrt {a_{t} } x_{t - 1} ,\left( {1 - a_{t} } \right)I} \right)$$

where the hyperparameters $a_{t}$ are pre-defined, small positive constants. The formulation can be reparametrized as follows:

4 $$q\left( {x_{t} {|}x_{0} } \right) = N\left( {x_{t} {|}\sqrt {r_{t} } x_{0} ,\left( {1 - r_{t} } \right)I} \right),\;q\left( {x_{t - 1} {|}x_{0} ,x_{t} } \right) = N\left( {x_{t - 1} {|}\mu ,\sigma^{2} I} \right)$$

where the process of removing noise produces a series of shape variables with different levels of noise, denoted as $x_{T} ,x_{T - 1} ,...,x_{0}$, where $x_{T}$ is sampled from a Gaussian prior and $x_{0}$ is the final output. The reverse process is conditioned on the conditioner, the incomplete point cloud $c$. Let $x_{T} \sim p_{laten}$ be a latent variable. The reverse process from latent $x_{T} { }$ to clean data $x_{0}$ is defined as

5 $$\begin{array}{*{20}c} {p_{\theta } \left( {x_{0:T} {|}x_{T} ,c} \right) = p\left( {x_{T} } \right){\Pi }_{t = 1}^{T} p_{\theta } \left( {x_{t - 1} {|}x_{t} ,c} \right)} \\ {p_{\theta } \left( {x_{t - 1} {|}x_{t} ,c} \right) = N\left( {x_{t - 1} ;\mu_{\theta } \left( {x_{t} ,{\text{c}},{\text{t}}} \right),\sigma^{2} {\rm I}} \right)} \\ \end{array}$$

where the mean $\mu_{\theta } \left( {x_{t} ,{\text{c}},{\text{t}}} \right)$ is a neural network that has $\theta$ as its parameters and the variance  $\sigma^{2}$ is a constant that depends on the time-step. To generate a sample that is conditioned on ${\text{c}}$, we first sample $x_{T}$ from a normal distribution, then we draw $x_{t - 1}$ from the conditional distribution $p_{\theta } \left( {x_{t - 1} {|}x_{t} ,c} \right)$ for each t = T, T − 1,…, 1, and finally we output $x_{0}$.

The goal of training the reverse diffusion process is to maximize the log-likelihood of the point cloud: ${\mathbb{E}}\left[ {\log {\text{p}}\left( {{\text{X}}\left( 0 \right)} \right)} \right]$. However, since optimizing the exact log-likelihood directly is intractable, we instead maximize its evidence lower bound (ELOB):

6 $${\text{L}}\left( \theta \right) = { }{\mathbb{E}}\left[ {\log p_{\theta } \left( {X^{0} } \right)} \right] \ge { }{\mathbb{E}}_{{q\left( {x_{0:T} } \right)}} \left[ {\log \frac{{p_{\theta } \left( {x_{0:T} } \right)}}{{q\left( {x_{1:T} |x_{0} } \right)}}} \right]$$

### Partition attention sampling

To gather local features efficiently and effectively, we propose a partition attention sampling (PAS) module. This module performs a subsampling operation on the input point cloud and passes the input features from the original points to the subsampled points. Other pooling methods employ a combination of sampling and query techniques. In the stage of sampling, points that will be used for the subsequent stage of encoding are sampled by using either farthest point sampling or grid sampling^31^. For each sampled point, a neighbor query is carried out to collect information from the points that are close to it. In these traditional sampling procedures, the query sets of points are not spatially aligned due to the uncontrollable information density. To address this, we propose PAS module.

**Figure 5:**
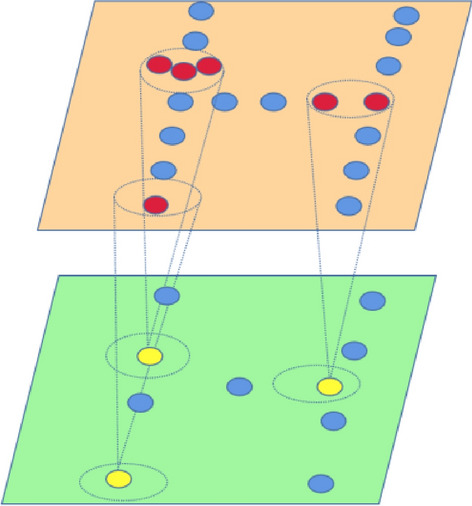
The attention-based partition sampling.

In the PAS module, we assume the input point set $S = \left( {P,F} \right)$, where P is the coordinate and F is the feature of the points. We partition S into subsets [$S_{1} ,S_{2} ,...,S_{n}$] by separating the space into non-overlapping partitions. We fuse each subset $S_{i}$ = ($P_{i}$, $F_{i}$) from a single partition as follows:

7 $$f_{i}^{\prime } = MaxPool\left( {Atten\left( {f_{i} } \right)} \right),\;p_{i}^{\prime } = {\text{MeanPool}}\left( {\left\{ {p_{j} } \right\}} \right)$$

where ($p_{i}^{\prime } ,f_{i}^{\prime }$) is the position and features of the pooling point aggregated form subset $S_{i}$, and Atten($\cdot$) is a self-attention layer. The PAS process is illustrated in Fig. 5. In our implementation, we choose k points in each partition, if the number of points is more than k points, we randomly select k points in each partition. If the points in each partition are less than k points, we repeat the center points $p_{i}^{\prime }$, until the total number is k. For the repeated points, we set the feature to zeros, so that the repeated points have no effect on the results. In Fig. 5, red points represent sampled points, and yellow points represent sampled points after sampling. Then we get the sampled points set $S^{\prime } = \left( {P^{\prime } ,\;F^{\prime } } \right)$. This sampling strategy not only reduces the parameters of the model but also ensures the generated point clouds meet the desired quality requirements.

### Muti-scale Feedback Refine Network

**Figure 6:**
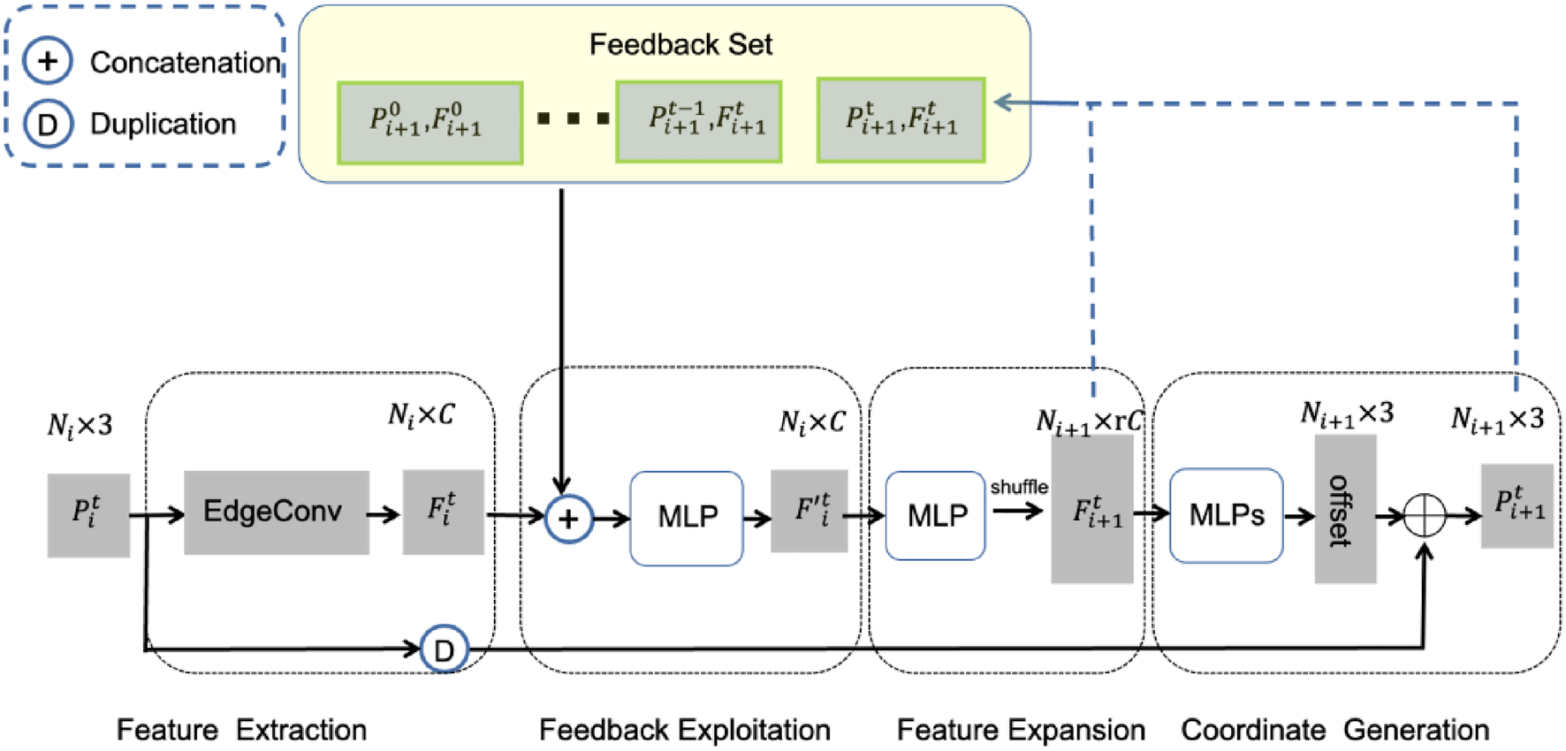
The detailed structure of the Multi-scale feedback refine network.

The obvious drawback of DDPM is the slow sampling speed, typically around 1000 steps in the generation process, which results in a very low generation efficiency despite its good quality and diversity. To solve this problem, we propose a Multi-scale Feedback Refine (MSFR) network to reduce the number of sample steps in the diffusion stage and use the MSFR to optimize the generated coarse point cloud to improve the generation speed. In particular, we use a feedback mechanism to train a refined network, to refine the coarse point cloud and to accelerate the model generation speed. In our work, the resolutions of high-layer feature maps can align with lower ones strictly and easily, and the high-resolution point features are transmitted back to enrich low-resolution point features. The detailed structure of MSFR is shown in Fig. 6, which consists of four parts: feature extraction, feedback exploitation, feature expansion, and coordinate generation. We first use EdgeConv^36^ to extract local geometric features ${F}_{i}^{t}$ from ${P}_{i}$. Then, a Multilayer Perceptron fuses present features ${F}_{i}^{t}$ with feedback information generated at the last step. Subsequently, the refined ${F}_{i}^{t}$ is expanded r times and then the order is shuffled. Note the coarse point cloud generated by the Conditional Generation Network as $U$. The predicted displacement is added to $U$ to obtain the refined point cloud $V$:$v=u+rf(u,c)$ where $v,u,c$ are the concatenated 3D coordinates of the point clouds $V,U,C$, respectively. $f$ is the MSFR Network, and $r$ is a small constant. In our experiment, we set it to 8. We use the CD loss between the refined point cloud $V$ and ground truth point cloud $X$ to supervise the network $\epsilon$. Throughout the training process of the MSFR network, the parameters of the conditional diffusion generation network are maintained at a constant value, after which we pre-generate and store the coarse point clouds in advance. Overall, our MSFR network effectively addresses the slow sampling speed issue of DDPM, enabling faster and more efficient generation while maintaining high-quality results.

## Data Availability

The datasets analyzed during the current study are available from the corresponding author upon reasonable request.
